# Improving Water Permeability of Hydrophilic PVDF Membrane Prepared via Blending with Organic and Inorganic Additives for Humic Acid Separation

**DOI:** 10.3390/molecules24224099

**Published:** 2019-11-13

**Authors:** Nasrul Arahman, Sri Mulyati, Afrillia Fahrina, Syawaliah Muchtar, Mukramah Yusuf, Ryosuke Takagi, Hideto Matsuyama, Nik Abdul Hadi Nordin, Muhammad Roil Bilad

**Affiliations:** 1 Department of Chemical Engineering, Universitas Syiah Kuala, Banda Aceh 23111, Indonesia; sri.mulyati@unsyiah.ac.id (S.M.); syawaliah2009@gmail.com (S.M.); mukramah@mhs.unsyiah.ac.id (M.Y.); 2 Graduate School of Environmental Management, Universitas Syiah Kuala, Banda Aceh 23111, Indonesia; 3 Graduate School of Engineering, Universitas Syiah Kuala, Banda Aceh 23111, Indonesia; afrilliafahrina26@gmail.com; 4 Center for Membrane and Film Technology, Kobe University, Rokkodai-Cho 1-1, Nadaku, Kobe 657-8501, Japan; takagi@harbor.kobe-u.ac.jp (R.T.); matuyama@kobe-u.ac.jp (H.M.); 5 Department of Chemical Engineering, Universiti Teknologi Petronas, Perak 32610, Malaysia; nahadi.sapiaa@utp.edu.my (N.A.H.N.); mroil.bilad@utp.edu.my (M.R.B.)

**Keywords:** polyvinylidene fluoride (PVDF), 2-methacryloyloxyethyl phosphorylcholine (MPC), polyvinylpyrrolidone (PVP), organic and inorganic additives

## Abstract

The removal of impurities from water or wastewater by the membrane filtration process has become more reliable due to good hydraulic performance and high permeate quality. The filterability of the membrane can be improved by having a material with a specific pore structure and good hydrophilic properties. This work aims at preparing a polyvinylidene fluoride (PVDF) membrane incorporated with phospholipid in the form of a 2-methacryloyloxyethyl phosphorylcholine, polymeric additive in the form of polyvinylpyrrolidone, and its combination with inorganic nanosilica from a renewable source derived from bagasse. The resulting membrane morphologies were analyzed by using scanning electron microscopy. Furthermore, atomic force microscopy was performed to analyze the membrane surface roughness. The chemical compositions of the resulting membranes were identified using Fourier transform infrared. A lab-scale cross-flow filtration system module was used to evaluate the membrane’s hydraulic and separation performance by the filtration of humic acid (HA) solution as the model contaminant. Results showed that the additives improved the membrane surface hydrophilicity. All modified membranes also showed up to five times higher water permeability than the pristine PVDF, thanks to the improved structure. Additionally, all membrane samples showed HA rejections of 75–90%.

## 1. Introduction

Membrane material properties play important roles in defining the performances of a filtration process. The morphology, pore size and distribution, hydrophilicity, and mechanical strength are considered as important intrinsic membrane properties. These membrane properties can be tuned through optimizing fabrication parameters via membrane material developments.

Polymers are commonly used as the base material for phase inverted membranes. The phase inversion process is the most popular method for membrane fabrication due to its simplicity and ability to produce variable membrane types and properties [1]. Some polymers widely used for membrane fabrications are polyvinylidene fluoride (PVDF), polyethersulfone (PES), polysulfone (PSF), and polyacrylonitrile (PAN) [2,3,4]. Among these polymers, PVDF offers the advantages of having good mechanical properties, good thermal stability, and excellent chemical resistance [2,5,6,7,8,9,10]. However, PVDF polymers are hydrophobic in nature, which promotes the affinity of organic molecules present in the feed solution on its surface and promotes membrane fouling or plugging in the membrane pores. Therefore, modifications in the fabrication method of PVDF membranes are required to tackle this issue.

Modifying the compositions of the dope solution for membrane fabrication by the presence of polymeric or inorganic additives is one of the most attractive methods to enhance the hydrophilicity of the resulting PVDF membranes. Some of the most common types of additives are made from polymers and inorganic particles. Polymeric additives act as copolymers to enhance membrane density, hydrophilicity, and also as pore forming agents [1,11]. One of the chemical additives that can potentially improve the hydrophilic properties of the PVDF membrane is the phospholipid polymers such as 2-(methacryloyloxy) ethyl phosphoryl chloline (MPC). This has been widely applied to reduce the absorption of proteins in the pharmaceutical field [12,13,14], but less so for membrane fabrication. MPC has the potential to enhance the hydrophilic nature of the PVDF membrane and can be used as surface modifying agents to increase the anti-fouling property of the resulting membrane [15,16].

Polyvinylpyrrolidone (PVP) is a more popular polymeric additive used for the fabrication of ultrafiltration (UF) or nanofiltration (NF) membranes. The PVP impacts on the resulting membranes’ structure and chemistry have been detailed elsewhere [17,18,19,20]. The morphological changes of a PVDF hollow fiber membrane made from a dope solution containing several concentrations of PVP have been investigated [21]. Increasing the concentration of PVP enhances the sponge-like structure across the membrane and reduces the number and size of the macrovoids. Increasing the PVP concentration from 6 wt% to 15 wt% also enhances the resulting membrane porosity [21]. Furthermore, the PVP modified PVDF membranes show increased hydraulic performance for the treatment of wastewater in membrane bioreactors [22]. The addition of 6 wt% PVP in the dope solution increased the water flux and reduced the membrane fouling rate for activated sludge filtration in a membrane bioreactor.

In addition to polymeric additives, inorganic particles are also often included as dope solution additives to improve the characteristics and performance of PVDF membranes [11]. The presence of inorganic particles in the membrane matrix has been examined to produce a membrane with high performance and properties. These are particularly applied as additives to enhance the mass transfer in pervaporation, to increase selectivity in gas separation applications, to improve hydrophilicity and fouling resistance properties, and to improve the mechanical properties [11,23]. They promote the formation of membranes with a more uniform pore distribution, better hydrophilicity, and improve the mechanical properties [24].

The most common inorganic particles are TiO_2_, SiO_2_, CNT, HNTs, and Al_2_O_3_ [5,11]. Among them, nanosilica (SiO_2_) has high potential due to its capacity to form the OH-bonds that induce hydrophilicity, low synthesis costs, and low toxicity in aqueous systems. Nanosilica is considered as an excellent additive to fabricate super-hydrophilic films, which are beneficial in mitigating membrane fouling [25], especially because it can be derived from sustainable sources such as bagasse. Lin et al. enhanced the performance of the PES membrane as demonstrated by enhanced permeability and better membrane fouling resistance by adding 0.3% of nano Stober silica in the dope solution [26]. In another study, SiO_2_ nanoparticles were incorporated in the PES membrane dope solution and better hydrophilicity and lower fouling propensity was found on the resulting PES/SiO_2_ membrane [25].

The effect of PVP or MPC concentration on the characteristics of the resulting membranes and their hydraulic performances has been widely reported [5,27,28]. Likewise, the effect of the SiO_2_ inorganic additive on the morphology of PVDF membranes is well understood. However, to our best knowledge, there has been no study on the effect of the combination of PVP or MPC with SiO_2_ on the characteristics and hydraulic performance of PVDF-based membranes. In this study, a systematic study on the impact of PVP, MPC as the sole additive, and their combination with SiO_2_ for the fabrication of PVDF-based membranes is reported. The SiO_2_ derived from bagasse was the renewable source is used. After fabrication, the morphology and surface chemistry of the resulting membranes were examined. Later, the hydraulic performances of the membranes were evaluated and its rejection performance was assessed by the filtration of humic acid (HA) solution.

## 2. Experimental

### 2.1. Material

Commercial PVDF with a molecular weight of 534,000 kDa was purchased from Sigma Aldrich (Sigma Aldrich, Co, Missouri, STL, USA). An organic solvent, dimethyl sulfoxide (DMSO), with an analytical purity of 99% was purchased from Merck KGAa, (Merck KGAa, Darmstadt, Germany). Two polymeric additives, MPC, and PVP (40 kDa) were purchased from Sigma Aldrich (Sigma Aldrich, Co). SiO_2_ nanoparticles were synthesized in-house from sugarcane bagasse. A non-woven support layer used as the baking material for membrane casting was donated by the Center for Film and Membrane Technology, Kobe University, Kobe, Japan.

### 2.2. Membrane Preparation

All membranes were prepared via a non-solvent induced phase separation method using the dope solution composition detailed in Table 1. A total of 20 wt% of PVDF polymer was dissolved in DMSO and 2 wt% of MPC and PVP as the copolymer was added separately into the dope solutions. The amount of the added SiO_2_ was 0.5 wt%. The high concentration of the PVDF was preferred to achieve a membrane with low pore size and has the capability to reject HA used as the feed solution in this study. The mixture of solvent, polymer, and additive was agitated at 80 °C until it reached a homogeneous condition and was kept for 24 h to release air bubbles. The dope solution was then cast onto a non-woven support using a hand-casting knife (applicator Yoshimitsu, Japan (YBA-4)) with a wet thickness setting of 200 µm. Membrane solidification was reached by immersing the casting plate into a batch containing the deionized water used as the non-solvent. The membrane sheet was stored in distilled water to completely release any residual solvent. It is worth noting that for the present study, the focus was mainly on the effect of the dope combination. The detailed optimization of the fabrication parameters will be conducted in a future study.

The preparation of the SiO_2_ used in this study was as follows. First, sugarcane bagasse was resized into small pieces. The isolation of silica from bagasse was conducted through extraction using a 10% KOH solution. The sugarcane bagasse was dissolved in the KOH solution at a weight ratio of 1:17 at boiling temperature for 1 h. To accelerate the settling, centrifugation at a rate of 2800 rpm was applied to the mixture for 30 min. At the final stage, the precipitate was dried and the silica particles were resized using ball mills.

### 2.3. Analysis of Membrane Morphology

The morphologies of the surface and cross-section of the membranes were observed using scanning electron microscopy (SEM, JSM-7500F, JEOL Ltd., Tokyo, Japan). To prevent excessive stress, the sample for cross-section was cracked after a series of treatment. It was first immersed in liquid nitrogen followed by freeze-drying (Eyela, EDU-1200, Tokyo, Japan) at a temperature of −55 °C. Before analyzing, all samples were sputtered by an osmium coater (Neoc-STB, Meiwafosis Co. Ltd., Tokyo, Japan) to enhance their conductivity. Surface roughness (Ra) was analyzed using atomic force microscopy (AFM; SII NanoTechnology, Inc., Tokyo, Japan, SPA400). After freeze drying (Eyela, EDU-1200), the uncoated sample was placed on the sample holder. The AFM image capture was supported by a microcantilever (SI-DF40), with a scan area of 1 × 1 mm. The average surface roughness value was obtained from processing of five image data by using the Spicel32 software.

### 2.4. Water Contact Angle

A water contact angle (WCA) meter (Drop Master 300, Kyowa Kaiwenkagaku, Saitama, Japan, CA-A) was used to analyze the surface hydrophilicity of the resulting membrane. A dried sample (2 cm × 0.5 cm) was attached tightly on the glass panel by using plastic double sided tape. 1 µL water was dropped on the top surface of the membrane by using a microneedle. The angle of the water drop was automatically recorded by the apparatus. The average water contact angle of each sample was collected from ten measurements at different areas of the membrane.

### 2.5. Chemical Composition

For identification of the surface chemical composition, the samples were first dried in an oven (Isuzu, SSR-115, Isuzu Seisakusho Co., Niigata, Japan) for three hours. Fourier transform infrared (FTIR) spectroscopy (Thermo Scientific iD5 ATR-Nicolet iS5 FTIR Spectrophotometer, Thermo Fisher Scientific, Waltham, MA, USA). was used to analyze the chemical compounds of the membrane surface. The IR spectra were recorded at a wavelength range of 400–4000 cm^−1^. The IR results were then processed using Omnic software (Thermo Fisher Scientific) to identify the chemical bonds.

### 2.6. Filtration Performance

**Figure 1:**
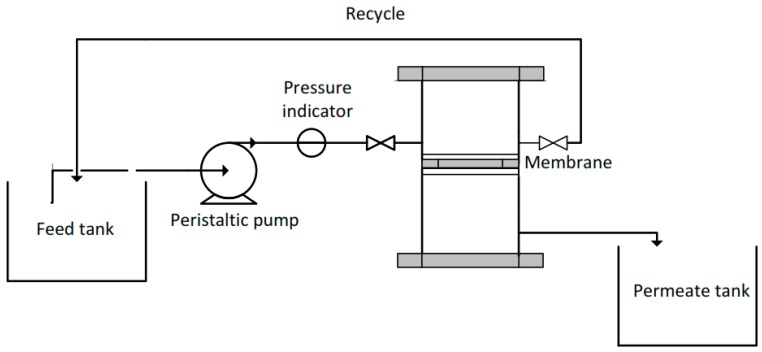
Experimental set up of the filtration system.

The illustration of the experimental set up is shown in Figure 1. The water permeability test was run using the crossflow-filtration mode. The cell was mounted with a flat sheet membrane with an effective filtration area of 9.075 cm^2^. The filtrations were conducted at room temperature and at a constant trans membrane pressure (TMP) of 1 bar. The feed solution was pumped through the filtration cell membrane using a peristaltic pump (Sci-323, Watson Marlow, Falmouth Cornwall, England). The water permeability (Wp) of the filtration was calculated using Equation (1).

(1) $$\mathrm{Wp}\;=\;\frac{V}{A.t.P}$$

where V is the permeation volume (L); A is the membrane surface area (m^2^); P is the applied pressure (bar); and t is the filtration time (h). The filtration experiments were run continuously and the permeate volumes were collected every 10 min until reaching a constant value, which was treated as the steady-state Wp reported in this study.

In terms of filtration performances, further tests were carried out to study the water contaminant rejection by using a humic acid (HA) solution of 10 ppm as the feed. Filtrations were run with the same module and the procedure for calculation of the Wp was according to Equation (1). The concentration of HA in the feed and the permeate was analyzed by using a spectrophotometer (Spectrometer UV-Vis 1800, Shimadzu, Japan), and the rejection of HA solution filtered by the membrane was calculated using Equation (2).

(2) $$\mathrm{HA}\;\mathrm{rejection}\;(\%)=\frac{C_{f}-C_{p}}{C_{f}}\times 100\;\%$$

where $C_{f}$ and $C_{p}$ is the concentration of the HA solution in the feed and the permeate, respectively.

## 3. Result and Discussion

### 3.1. Membrane Morphology

**Figure 2:**
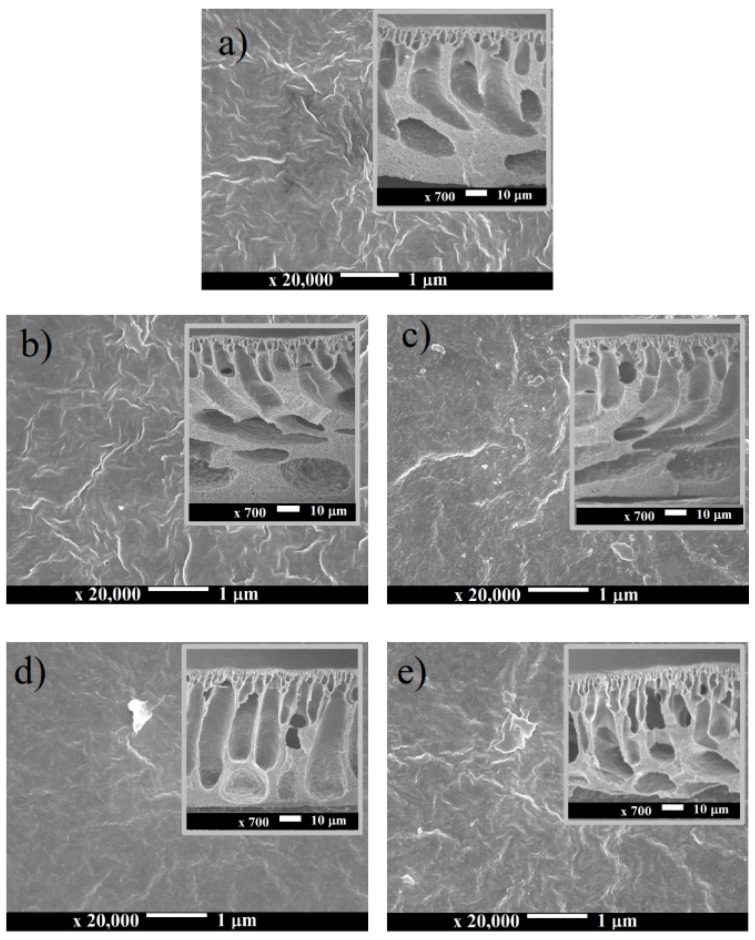
The microstructure SEM image of the morphology of the membrane surface and cross-section (in the inset) of pristine (**a**): F0, and modified membrane (**b**) FPc; (**c**) FPc-Si; (**d**) FVp; (**e**) FPv-Si.

The morphologies of the resulting PVDF membranes were strongly affected by the presence of the additive in the dope solution as demonstrated in Figure 2. Such properties were strongly related with other membrane characteristics as well as the filtration performance. SEM images of the top surface and its cross-section view for pristine and all PVDF membrane samples are depicted in Figure 2. The SEM images show that all membranes had dense surface structures, most likely in the pore size range of an ultrafiltration membrane. However, no visible pores appeared on the membrane surface because of the resolution limitation of the surface SEM images. The dense top layer structure was the result of a high PVDF concentration (of 20 wt%) in the dope solution. The surface pore of the membrane fabricated via the wet inversion method was affected by the polymer concentration in the dope solution. Application of a lower PVDF concentration may lead to the formation of a PVDF membrane with a larger pore size, as described elsewhere [29,30].

Figure 2 also shows the cross-section SEM images of the membrane samples. All membrane samples had asymmetric structure with the irregular shape of macrovoids supported by sponge-like structures in the bottom layer. It is worth pointing out that the sponge-like structure at the bottom layer of the F0 membrane was larger than the rest. The FPv membrane had a different morphology from other membranes, which constituted a finger-like macrovoid structure with a regular shape extending from underneath the top skin to the bottom skin layer.

**Figure 3:**
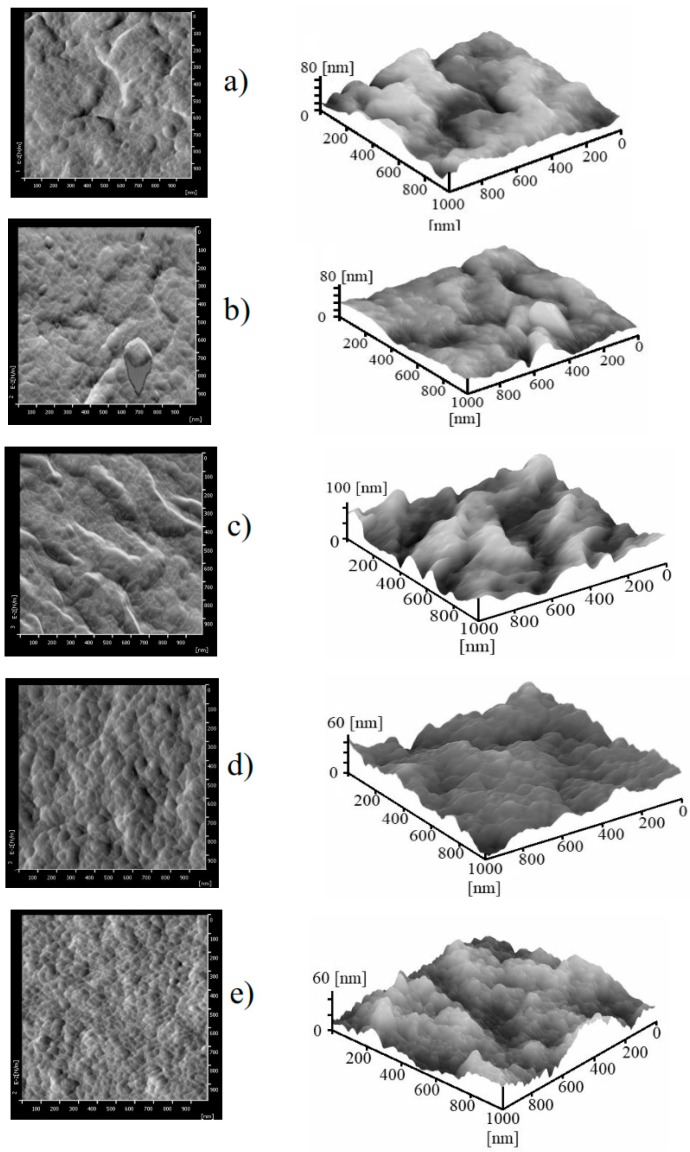
The 2D, and 3D AFM images of the surface of pristine (**a**) F0 and modified membranes (**b**) FPc; (**c**) FPc-Si; (**d**) FVp; (**e**) FPv-Si.

The 2D and 3D images of the AFM measurements for all membrane samples are depicted in Figure 3. Detailed information on the nanoscale surface morphology of the membrane can be extracted from the AFM images. The quantitative parameter on the surface morphology from AFM was interpreted using the surface roughness (Ra) parameter. The Ra also quantifies the nodule-like structure forming peaks and valleys on the surface of the membrane. Moderate peaks and valley patterns were formed on the surface of the F0 and the FPc membranes with Ra values of 11.39 and 10.44 nm, respectively. The findings imply that the addition of MPC and SiO_2_ into the dope solutions results in membranes with a high surface roughness, as shown in Figure 3 (for FPc-Si). The Ra of the FPc-Si membrane was 18.25 nm. On the other hand, more uniform and smaller nodule patterns existed on the FPv membrane. The combination of PVP and SiO_2_ in the FPV-Si membrane yielded a surface structure with the Ra value of 6.71 nm, slightly lower than the Ra of the FPv membrane of 9.58 nm that used solely PVP as the additive.

### 3.2. Hydrophilicity

**Figure 4:**
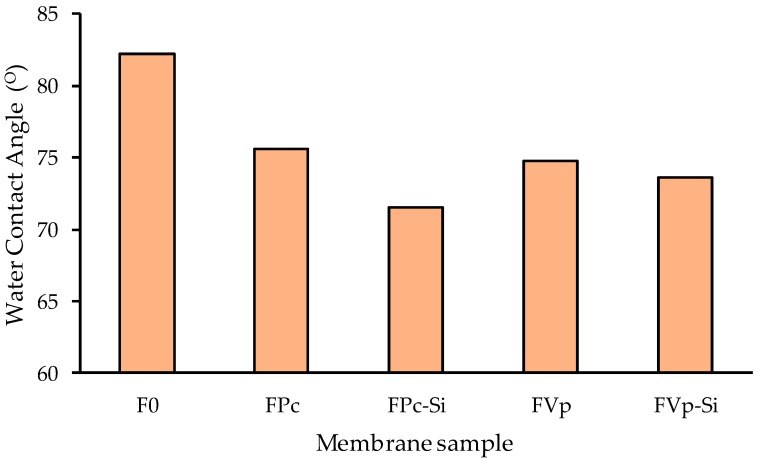
The hydrophilicity profile of all membranes measured by using the water contact angle meter.

The hydrophilicity of the prepared membranes was evaluated through the WCA values as shown in Figure 4. It shows that the incorporation of additives in the dope solutions successfully increased the membrane surfaces’ hydrophilicity of the PVDF as reflected by the lower WCA values. The pristine PVDF showed the highest contact angle of ~81° and upon additions of either PVP or MPC; the WCA decreased to ~75 °C. The WCA for the PVDF membrane prepared by the dry/wet method, as applied in the current study, was often lower than 90° [31], despite being prepared from a PVDF polymer with a low surface tension because of the flat nature of the resulting membrane [32,33]. PVP is an additive widely known and appreciated for its hydrophilic characteristic [34,35,36]. Moreover, MPC has also been reported to have good interactions with water due to its zwitterionic properties and thus induces its hydrophilic property [27]. Figure 4 also shows that the addition of silica also decreased the WCAs for both PVP-bended and MPC-blended membranes thanks to the hydrophilic nature of the silica [37]. When the PVDF polymer was mixed with either PVP or MPC in the dope solutions, more abundant hydrophilic functional groups remained on the membrane matrices to impose their hydrophilic property. It is worth pointing out that the WCA of the FPc-Si was slightly lower than the FVp-Si, most likely because higher residual additives remained in the membrane matrix, as shown by the high intensity of C=O FTIR peaks in Figure 6.

### 3.3. Membrane Surface Chemistry

**Figure 5:**
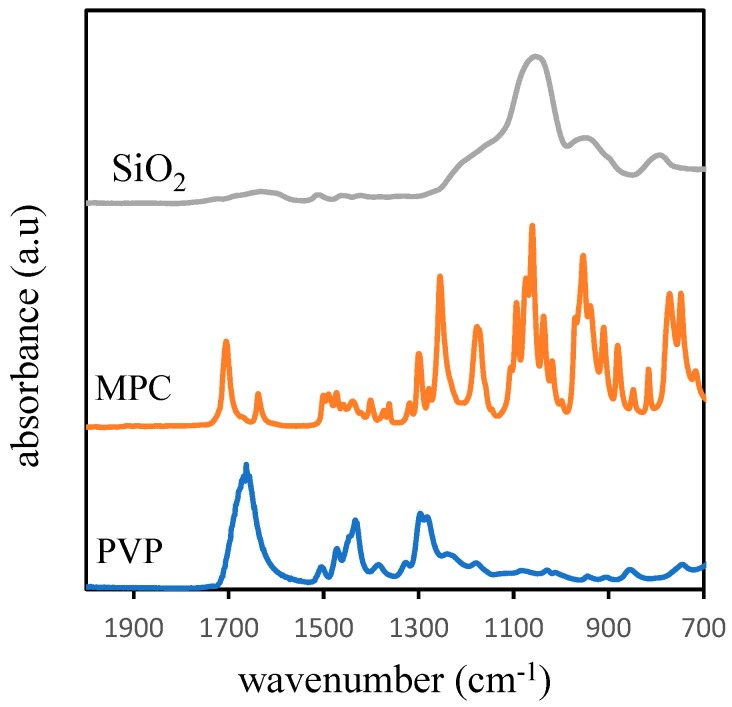
The FTIR spectra of the additives used for membrane fabrication.

**Figure 6:**
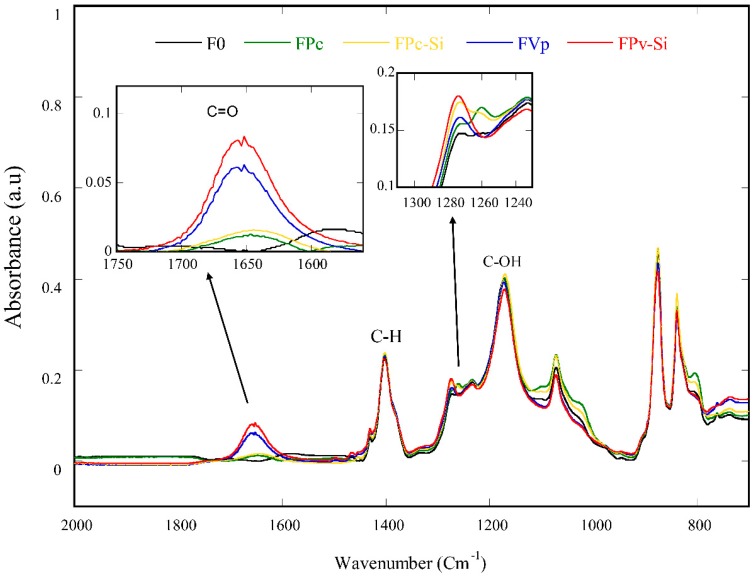
FTIR spectra of the membrane samples.

Figure 5 and Figure 6 reveal the IR spectra of the MMA used as the additive for the dope solution preparation and the resulting membranes, respectively. Generally, the IR spectra were dominated by functional groups of PVDF polymer denoted by wide peaks around 865, 1180, and 1407 cm^−1^. The characteristic of the PVDF polymer appears at a peak of 1407 cm^−1^, corresponding to the deformation vibration of the asymmetric CH_2_ functional group and at a peak of 1180 cm^−1^, corresponding to the CF_2_ functional group [8].

The presence of PVP displayed on the IR spectra at a peak of 1665 cm^−1^ was assigned to the carbonyl groups (C=O), as shown in Figure 5 and Figure 6. The characteristic of MPC can be ascribed by the presence of a peak at wavenumber 1260 cm^−1^, associated with C−O stretching. The adsorption of silica as an inorganic additive is represented by a weak peak at the wavelength of 1263 cm^−1^. The weak nature of the peak may be due to a low concentration of the silica present in the membrane matrix.

### 3.4. Filtration Profile

**Figure 7:**
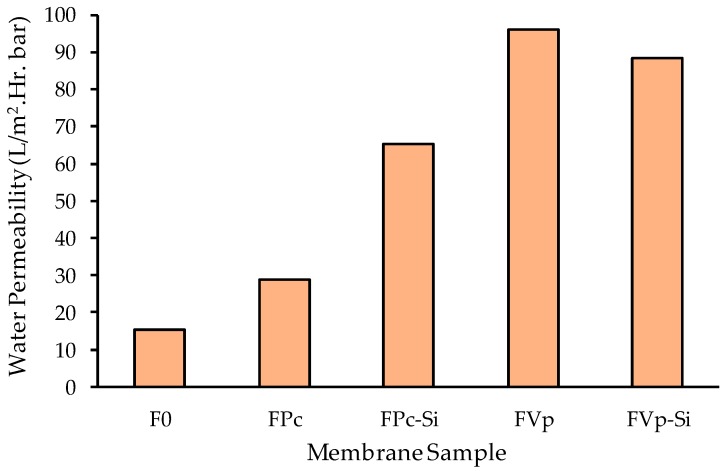
Pure water permeability of the membrane samples.

Figure 7 shows that all modified PVDF membranes had higher water permeability than the pristine PVDF membrane used as the reference. It is worth noting that the water permeability of the FPv was much higher than the FPc, indicating the advantage of PVP as a more desirable additive than the MPC in enhancing hydraulic performance. The results confirm the preference of employing PVP as a pore forming agent in membrane fabrication [22].

The addition of both MPC and SiO_2_ simultaneously into the dope solution to form the FPc-Si membrane led to almost double the water permeability than that of the sole addition of MPC (in the FPc membrane). Both MPC and SiO_2_ are hydrophilic compounds and contribute positively to enhance the exchange rate of the solvent and non-solvent during the phase inversion process. Rapid/instantaneous demixing leads to the formation of a membrane with thin top layer and less dense membrane [25,37]. The addition of the SiO_2_ on top of the presence of MPC in the dope solution lead to faster demixing. Despite contributing to enhancing the solution viscosity that normally decreases the demixing rate, the addition of Si still within the amount can favor hastening the demixing process, thanks to its hydrophilic property. At higher Si and MPC loadings, one can expect the opposite role of the additive in slowing down the demixing rate.

Unlike in FPc-Si, the water permeability of the FPV-Si is slightly lower than the FPv (Figure 7). As detailed in Table 1, the composition of the dope solution for FPv-Si is from the dope solution of FPv and addition of SiO_2_. The PVP additive is a hydrophilic polymer with a large molecular weight with strong impact on increasing the dope solution viscosity. Hence, the addition of SiO_2_ into the dope solution containing PVP further increases the solution viscosity. Under this condition, the kinetic effect dominates the phase inversion process. High dope solution viscosity leads to a lower rate of solvent–nonsolvent exchange in the coagulation bath, which results in a denser membrane with lower hydraulic performance. A membrane with low porosity has lower clean water permeability as reported earlier [11,26]. The morphology of the FPV-Si membrane exhibited in Figure 2 also proves that macrovoid geometry in the FPv-Si membrane is narrower than the ones in the FPv membrane.

**Figure 8:**
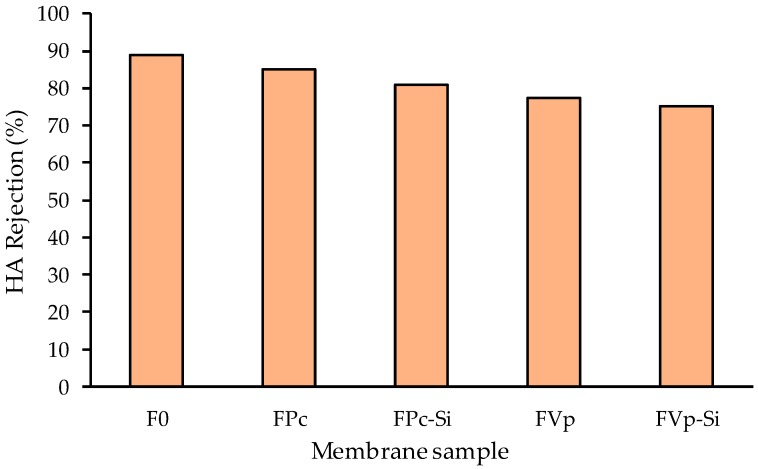
Humic acid rejection of the membrane samples.

**Figure 9:**
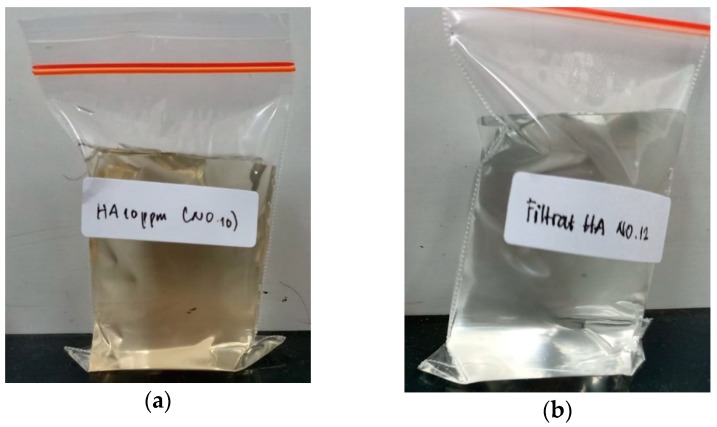
The appearance of the feed humic acid solution (10 ppm) (**a**) and the permeate after filtration by the FPV-Si membrane (**b**).

Increasing the water permeability as the result of the incorporation of additives does not significantly affect the HA rejection in the feed solution of 10 ppm HA solution, as shown in Figure 8. The rejection of HA by all modified PVDF membranes ranged from −75% to 90%. Such rejections were also reflected by the appearances of the feed and permeate solutions of the FPv-Si membrane displayed in Figure 9. The rejection values were considered to be high when considering the low concentration of HA in the feed solution (10 ppm). Higher rejections are expected when treating a solution with a higher HA concentration.

## 4. Conclusions

The addition of hydrophilic additives in the dope solution of the PVDF-DMSA system alters the resulting membrane morphology by increasing the Ra, especially for FPc-Si and FPv-Si. It also enlarges the macrovoid size for FPc, FPc-Si, FVp, and FPv-Si, and turns the cross-section structure of the FPv-Si into finger-like morphology. The presence of residual additives on the membrane matrix, as shown by the IR spectra, improves the membrane surface hydrophilicity indicated by a lower WCA. The impact is most profound for the FPc-Si membrane ascribed by the intense FTIR peak of C=O on the FPc-Si membrane. All of the modified membranes showed a higher pure water permeability than the pristine PVDF thanks to the improved structure and more hydrophilic surface chemistry. Additionally, all membrane samples showed HA rejections of 75–90%. Overall results suggest the efficacy of all additives in enhancing the hydraulic performance of the resulting membrane without significantly affecting HA rejection. Further study to explore the fabrication involving mixed additives, especially silica from a green source, is required to obtain the optimum membrane that offers high hydraulic performance coupled with high HA rejection.

## Figures and Tables

**Table 1:** Dope composition.

Code	Polymer Composition (wt%)				
	PVDF	MPC	PVP	SiO_2_	DMSO
F0	20				80
FPc	20	2			78
FPc-Si	20	2		0.5	77.5
FVp	20		2		78
FVp-Si	20		2	0.5	77.5
